# Room temperature blooming of CeO_2_ 3D nanoflowers under sonication and catalytic efficacy towards CO conversion†

**DOI:** 10.1039/d0ra02554b

**Published:** 2020-06-09

**Authors:** Deblina Majumder, Indranil Chakraborty, Kalyan Mandal

**Affiliations:** S. N. Bose National Centre for Basic Sciences Block JD, Sector III, Salt Lake Kolkata 700106 India deblina293@gmail.com

## Abstract

Carbon monoxide (CO), being a highly toxic gas, bears hazardous effects on human health and contributes majorly to environmental pollution. It is mostly produced by automobile exhausts and incomplete combustion of carbon-containing substances. Thus, the development of catalysts for CO conversion is highly imperative and has always gained interest for real field applications. Besides the high oxygen storage capacity and facile transitions between oxidation states, the huge abundance of cerium on earth makes CeO_2_ a low-cost and highly effective alternative to noble metal catalysts for CO oxidation. The present work delineates the room temperature synthesis of flower-shaped 3D CeO_2_ nanostructures using a sonication-assisted simple synthesis method within 2 hours under the pivotal importance of a structure-directing agent, polyvinylpyrrolidone (PVP). The bifunctional contributions of PVP as a surfactant and as a capping agent are discussed with a plausible mechanism. The method leading to the formation of hierarchical CeO_2_ nanoflowers provides an appreciable surface area of 132.69 cm^2^ g^−1^. The morphological and structural characterizations of the catalyst were thoroughly investigated using FESEM, TEM, XRD, UV-visible spectroscopy, photoluminescence spectroscopy, FTIR spectroscopy and X-ray photoelectron spectroscopy. The structural efficacies of flower-like CeO_2_ nanostructures have also been correlated to the narrowing of the band gap and the generation of the corresponding oxygen vacancies, resulting in surface catalytic properties towards 80% conversion of CO.

## Abstract 2

Carbon monoxide (CO), being a highly toxic gas, bears hazardous effects on human health and contributes majorly to environmental pollution.

## Graphical abstract

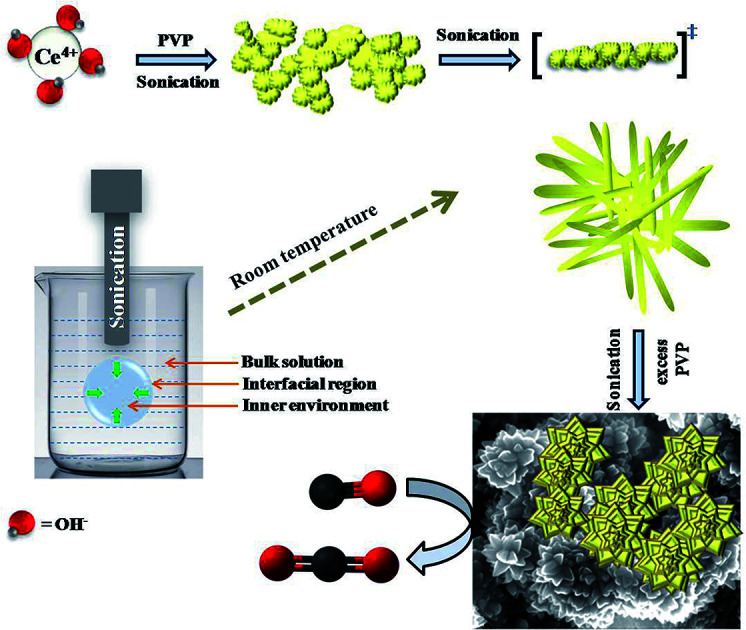

## 1. Introduction

The increasing production of carbon monoxide (CO) has become an undeniable part of daily life due to the enormous combustion of fossil fuels, automobile engines and industrial chemical activities.^1^ The environmental toxicity of CO is very well known. Its higher concentration in the atmosphere is hazardous to life as it readily binds with haemoglobin and forms carboxyhaemoglobin. As a consequence, the oxygen-carrying capacity of blood becomes effectively reduced, resulting in several illnesses, suffocation, and even death.^2^ The aim is, therefore, to convert CO into CO_2_, a less toxic, natural gas (exists in air) and also a feedstock for methanol production used to produce many chemical compounds that are valuable for human life, such as plastics.^3^ An effective catalyst is required for CO conversion. The catalytic converter materials used in automobile exhaust contain traces of highly precious metals such as gold, silver, palladium, platinum, and rhodium.^4,5^ These precious metals are mostly doped on support materials such as Fe_2_O_3_, TiO_2_, CeO_2_, Co_3_O_4_, and NiO.^6^ However, the synthesis and development of these oxide nanocomposites are highly expensive and they are prone to deactivation and deterioration, especially at high temperatures, due to the increase in mobility and sintering.^7^ Thus, catalysts made of bare oxide materials are still imperative for the oxidation of automobile exhaust, especially CO.^8,9^ Saalfrank *et al.* reported in detail the advantages and scope of the activity of noble metal-free oxide catalysts for CO oxidation.^10^ Many bare metal oxides are used for CO conversion, such as Co_3_O_4_, Cu_2_O, V_2_O_5_, SnO_2_, ZnO, NiO and CeO_2_.^11^ Although, the oxides of Co and Cu show appreciable activity towards CO oxidation, their usage in catalytic converters has been limited due to the high susceptibility of deactivation by water in ambient conditions.^11^ V_2_O_5_ is deactivated rapidly at higher concentrations of CO (even greater than 10% of v/v) due to the loss of active sites *via* the depletion of inter-lattice oxygen. SnO_2_ being susceptible to moisture and requiring high calcination temperatures often renders a significant decrease in catalyst activity due to the associated thermal aging, while the high activation temperature of ZnO limits the bare deployment of this oxide catalyst as well. The very high selectivity of NiO for oxygen as compared to CO restricts its scope for real field applications.^11^ In comparison to the abovementioned catalysts, researchers have demonstrated the pivotal role of CeO_2_ in the field of CO conversion mainly due to its high oxygen storage capacity, thermal stability and high affinity towards CO, *etc.*^12,13^ Cerium dioxide (CeO_2_), being abundantly available in the earth's crust, is a great choice as a semiconductor metal oxide for a wide variety of applications including catalysis, sensors, optoelectronics, photocatalytic materials, *etc.*^14^ CeO_2_ nanoparticles are peculiarly favourable owing to their high oxygen storage capacity and the change in oxidation state from Ce^3+^ to Ce^4+^. An inherent property that makes the versatile metal-oxide CeO_2_ even more special is its ability to morph into hierarchical superstructures. The creation of such exotic morphological features by tweaking growth parameters often renders tuned physicochemical properties that can be deployed for specific applications with respect to the surface area, catalytic properties, *etc.* In this respect, spherical or two-dimensional structures of CeO_2_ are perhaps the most widely explored variants of CeO_2_ nanoparticles. A comprehensive survey of diverse methods adopted for producing superstructures of CeO_2_ has been summarised well. Various methods such as solvothermal synthesis, co-precipitation,^15^ electrode deposition, hydrothermal, sol–gel and precipitation methods have been reported for providing control of the evolution of morphology, size, surface area, pore size, *etc.*^16–19^ Besides the above conventional ways, some other preparatory methods such as the reversed micelles route,^20^ forced hydrolysis,^21^ an electrochemical method,^22^ decomposition of oxalate precursors,^23^ urea-based or hexamethylene tetramine-based homogeneous precipitation,^24–27^ the use of hydrazine monohydrate,^28^ solvothermal and solid-state reactions,^29,30^ metal–organic chemical vapour deposition, spray pyrolysis have also been reported.^31^ In recent years, ultrasound and microwave irradiation have been extensively used to generate novel materials with unusual properties. Ultrasound irradiation can induce the formation of particles with a much smaller size and higher surface area than those reported by other methods.^32^ Zhang *et al.* reported for the first time the ultrasonic-assisted one-step synthesis of CeO_2_ nanorods *via* a simple liquid-phase synthesis method using PEG as a structure-directing agent.^33^ Apart from 1-D structures, distinct assemblies of two-dimensional (2-D) and hierarchical three-dimensional (3-D) structures have also grabbed special attention due to their specific attributes for enhanced application.^34^ While nanospheres and nanorods are the common form of nanoceria material superstructures, other types of morphologies, like nanoflakes, spindles and flowers, also exist.^35^ Besides the growing interest in the synthesis of one- or multi-dimensional CeO_2_ structures, it is also important to investigate facile techniques to develop these hierarchical structures. For example, changing the precursor concentration and optimization of additive and reaction parameters could exclusively act as influential factors in the formation of different structures as desired. In the literature, one- or multi-dimensional superstructures have been produced by modifying synthesis parameters, or through the hierarchical growth approach, where the desired morphology was achieved through the augmentation of an existing structure. The sonochemical method, deployed in the present study for implementing the structural growth, offers a range of advantages like the conservation of energy and faster kinetics. Additionally, the sonochemical technique facilitates the entire process occurring at room temperature, thereby rendering it as a green approach. An inexpensive room temperature synthesis technique is highly feasible towards obtaining a scalable approach. Motivated by the above facts, the current study details the synthesis of a flower-shaped CeO_2_ catalyst using cerium ammonium nitrate as the precursor and PVP as a structure-directing agent under ultrasonication at room temperature. Post-synthesis, the surface catalytic reactivity of CeO_2_ structures was also deployed toward CO conversion, keeping its toxicity in mind.

## 2. Experimental section

### 2.1. Materials

Ammonium cerium(iv) nitrate [(NH_4_)_2_Ce(NO_3_)_6_, ≥98.5%] was procured from Sigma-Aldrich. Polyvinylpyrrolidone [(PVP) ≥99.8%], isopropyl alcohol, ammonium hydroxide and ethylene glycol (EG) (anhydrous, HOCH_2_CH_2_OH, ≥99.8%) were purchased from Loba Chemie Pvt. Ltd., India. Deionized water was used for analytical purposes during the synthesis.

### 2.2. Synthesis method

Considering the equivalent molal ratio, 1 g of PVP was added to 100 mL of 0.009 mol% solution of (NH_4_)_2_Ce(NO_3_)_6_ in EG. The mixture was then vigorously stirred using a magnetic stirrer at the speed of 300 rpm for 2 h. Then, 10 mL 1 N NH_4_OH was gradually added to the reaction mixture with vigorous stirring during ultrasonication until the pH value was greater than 9. Sonication was continued for 2 h. After 45 minutes of sonication, the colloidal solution became turbid and was named Ce-A. With the elapse of sonication time, precipitation was observed after approximately 70 minutes. The stage of the first instance of distinguishable precipitation was named Ce-B. The sonication was then continued for 2 h with the addition of 0.15 g of PVP in total, and assuming the completion of precipitation, the final product was named Ce-C. For each case, the product was collected after centrifugation at 8000 rpm and washed with isopropyl alcohol until the litmus turned to violet. Finally, the collected samples were air-dried, followed by calcination at 500 °C for 5 h.

### 2.3. Characterization

An X-ray diffractometer (Bruker D8 Advance, Cu Kα line) was used for the phase analysis of the sample. Scanning transmission electron microscopy-energy dispersive spectroscopy (FEI QUANTA FEG) system and high-resolution transmission electron microscope (HRTEM: FEI TECNAI G2 F20-ST) were deployed for morphological analyses. The optical properties were analysed using a Shimadzu UV 2450, and the Brunauer–Emmett–Teller (BET) surface area was measured using Quantachrome Instruments, version 3.0. Photoluminescence (PL) spectroscopy was done using a Horiba Jobin Yvon Fluorolog fluorimeter by irradiating the sample at *λ*_ex_ = 320 nm. Fourier transform infrared spectroscopy (FTIR) measurements were carried out using a JASCO FTIR 6300. Raman spectroscopy was carried out using a laser source of 514 nm in a Renishaw System. The samples in powder form were pressed onto the surface of sticky carbon conductive tape for analysis using a Versa probe XPS model (base pressure was maintained at 6 × 10^−10^ mbar with an energy resolution of 0.6 eV). Mass spectroscopic results were obtained using liquid chromatography-mass spectrometry (Waters 2695, USA) spectrometer. The catalytic activity of the as-prepared material was carried away in a continuous flow based microreactor at atmospheric pressure. The analysis was carried out by placing 50 mg of catalyst nanoparticles in the reactor with a continuous flow rate of 30 sccm of CO (compressed ≥99%, molecular mass: 28 g mol^−1^, critical pressure: 3499 kPa, density = 1.2501 kg m^−3^ at 273 K; Praxair, Inc). Post catalysis, the results were analysed using a gas chromatograph (ClarussSQ 8 GC/MS). A temperature-dependent study was also performed, keeping other parameters unaltered.

## 3. Results and discussions

### 3.1. Crystallographic and morphological analysis

**Fig. 1:**
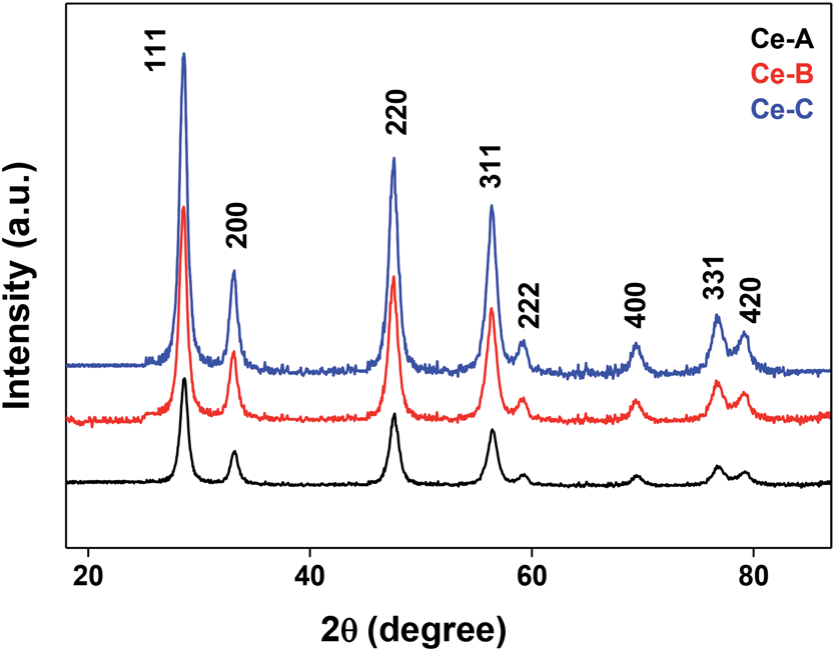
XRD patterns of different CeO_2_ nanomaterials.

**Fig. 2:**
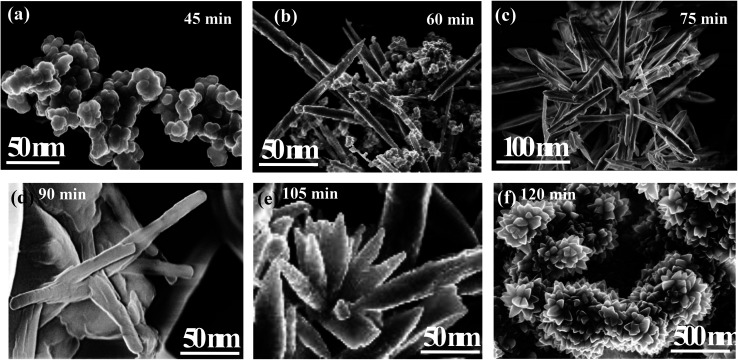
FESEM images of the morphological evolution of ceria nanoflowers: (a) Ce-A, a lump-like formation after 45 min of sonication; (b) the accumulation of lumps towards the formation of rod-like structures after 1 h of sonication; (c) Ce-B, monodisperse rod-like ceria after 75 min of sonication; (d) an intermediate stage showing the fusion of rods after 90 min of sonication; (e) post-PVP addition, fused petal formation after 105 min of sonication; (f) Ce-C, ceria nanoflowers after 2 h of sonication.

**Fig. 3:**
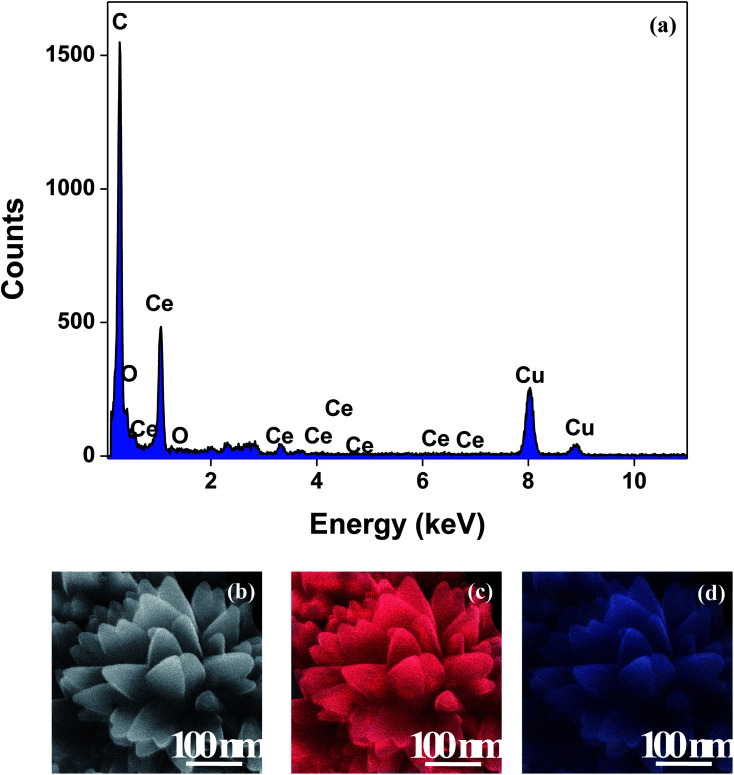
(a) TEM-EDAX plot; (b) representative FESEM images of a Ce-C ceria nanoflower and the corresponding FESEM-EDAX elemental mapping analysis for (c) Ce and (d) O.

**Fig. 4:**
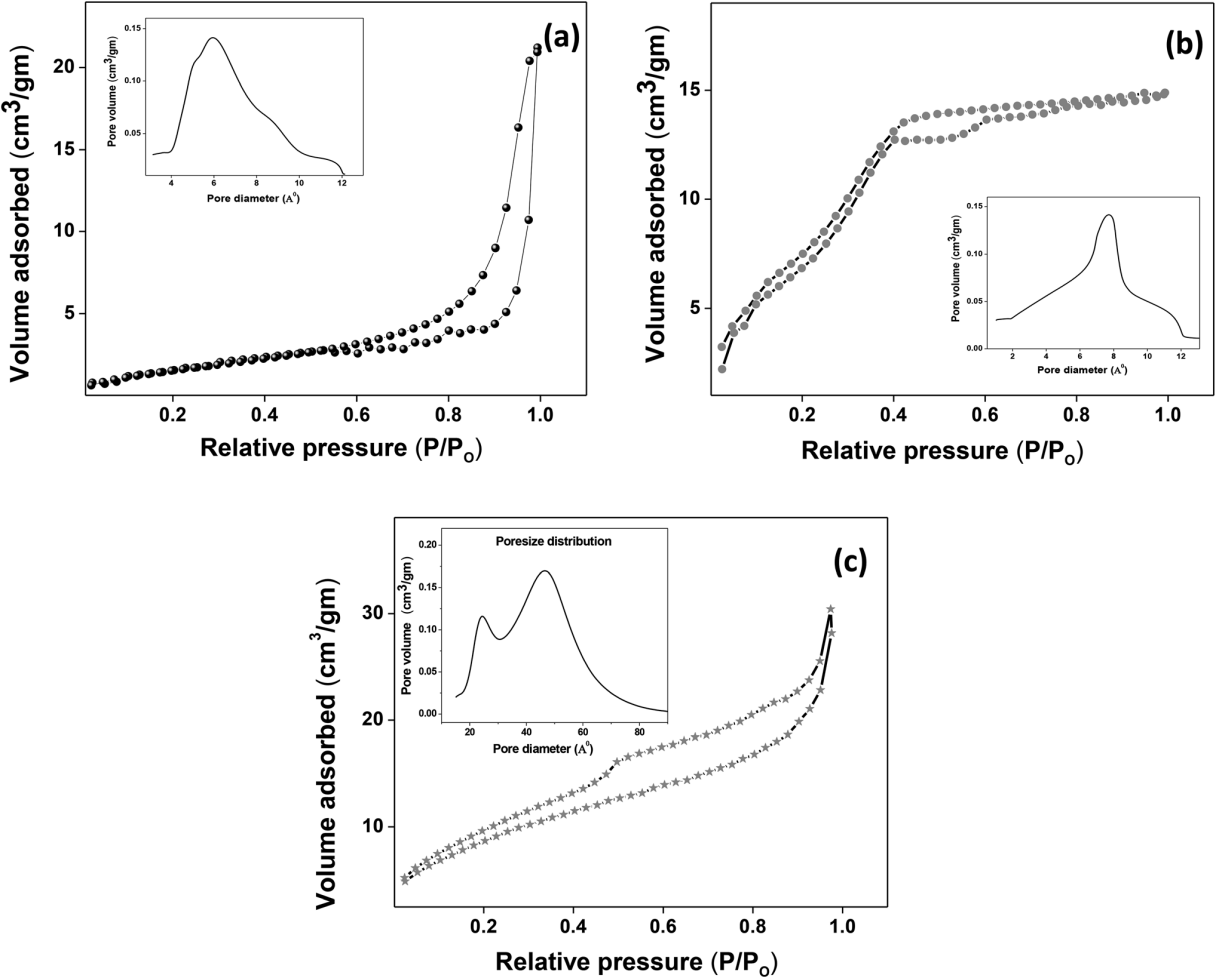
(a–c) Nitrogen adsorption/desorption isotherms of Ce-A, Ce-B and Ce-C, respectively, with pore-size distribution curves given in the insets.

The XRD crystallographic analysis of the obtained samples showed that the diffraction peaks in Fig. 1 correspond to the (111), (200), (220), (311), (222), (400), (331) and (420) planes, which can be assigned to the face-centered cubic structure for the anatase phase of CeO_2_ (space group *Fm*3*m*; lattice constant, *a* = 5.412 Å according to JCPDS 78-0694). Post-calcination XRD analysis is provided in Fig. S1.† The morphological evolution of CeO_2_ nanoparticles could be traced from their FESEM and TEM images, presented in Fig. 2 and S2,† respectively. Fig. 2(a) shows the formation of lump-like CeO_2_ nanoparticles, Ce-A (first colloidal appearance after 45 minutes of sonication during synthesis), while Fig. 2(b) represents the intermediate state (after 1 h) of formation of rod-like structures by the accumulation of Ce-A. After 75 minutes of sonication, monodisperse rod-shaped structures (Ce-B) seemed to be formed [shown in Fig. 2(c)]. The intermediate structural evolution was investigated by performing FESEM on the reaction aliquot at 15 minutes time intervals after the addition of excess PVP and the fusion of the rod-shape structures was observed [Fig. 2(d)]. After some time, the fused rod-like structures seemed to grow petals [Fig. 2(e)] and finally, after 2 h of sonication, hierarchical flower-like superstructures, evident from the corresponding FESEM in Fig. 2(d) were achieved. The TEM micrographs of Ce-A, Ce-B and Ce-C are included in Fig. S2† with the corresponding SAED pattern, HRTEM and FFT, where the bright spots and lattice fringes confirmed the crystalline nature of the materials. The significant differences in crystallinity among the three CeO_2_ samples were observed from their XRD measurements, where crystallinity is defined as the ratio of the intensity from the crystalline peaks to the sum of the crystalline and amorphous intensities; crystallinity = (total area of crystalline peaks)/(total area of all peaks) × 100. By following the above equation, the obtained crystallinity follows the order, Ce-C (89%) > Ce-B (72%) > Ce-C (66%). Rietveld analysis was performed for all the samples to obtain their structural parameters. The details are included in Table 1. The lattice constant is the side length of the cube (for cubic crystals), or side length for a hexagonal wurtzite crystal and the bond length is the distance between the nearest atoms, which is different for FCC, BCC or simple cubic crystals, even if they have the same lattice constant. Typically, smaller bond lengths mean that the electrons are more tightly bound to the atom, and hence require more energy to remove, leading to an increased bandgap. Reportedly, theoretical modelling assumes smaller lattice constants corresponding to higher bandgaps.^36^ In this work, increases in lattice constants and Ce–O bond lengths were observed. These could be assigned to the increase in the oxygen vacancy and narrowing in the band gap in ceria nanoflowers (Ce-C), and could easily be corroborated with their catalytic activity towards CO conversion.^37^ A detailed formation mechanism is discussed later. The chemical composition and distribution of the constituents were envisaged using elemental mapping on the Ce-C nanoflower. The corresponding EDAX analysis along with the mapping from a region of the material is furnished in Fig. 3, where Fig. 3(a) shows TEM-EDAX spectra, confirming the absence of any impurity, and Fig. 3(b)–(d) represent the homogeneous distribution of all the basic elements Ce and O throughout the petals of the nanoflowers. The above analyses outline the pictorial steps of formation, leading to the formation of a 3D hierarchical flower-like morphology that provides the accessible effective specific surface area with the feasible tuning of their properties. The mesoporosity of the 3D nanoflowers is evident as shown in Fig. 4(c) (inset). This contributes to the pivotal role of the appreciable effective surface area, facilitated during the catalytic conversion of CO gas.

**Table 1:** Structural parameters of CeO_2_ samples *via* Rietveld analysis

Structural parameters	Ce-A	Ce-B	Ce-C
Lattice constant, *a* (Å)	5.4137 (8)	5.4140 (8)	5.4142 (8)
Lattice constant, *b* (Å)	5.4137 (8)	5.4140 (8)	5.4142 (8)
Lattice constant, *c* (Å)	5.4137 (8)	5.4140 (8)	5.4142 (8)
Cell angle, *α*	90	90	90
Cell angle, *β*	90	90	90
Cell angle, *γ*	90	90	90
Cell volume (Å ^3^)	158.66 (4)	158.70 (4)	158.71 (4)
Ce–Ce bond length (Å)	3.8281 (8)	3.8283 (8)	3.8284 (8)
Ce–O bond length (Å)	2.3441 (4)	2.3442 (4)	2.3444 (4)

### 3.2. Spectroscopic analysis

**Fig. 5:**
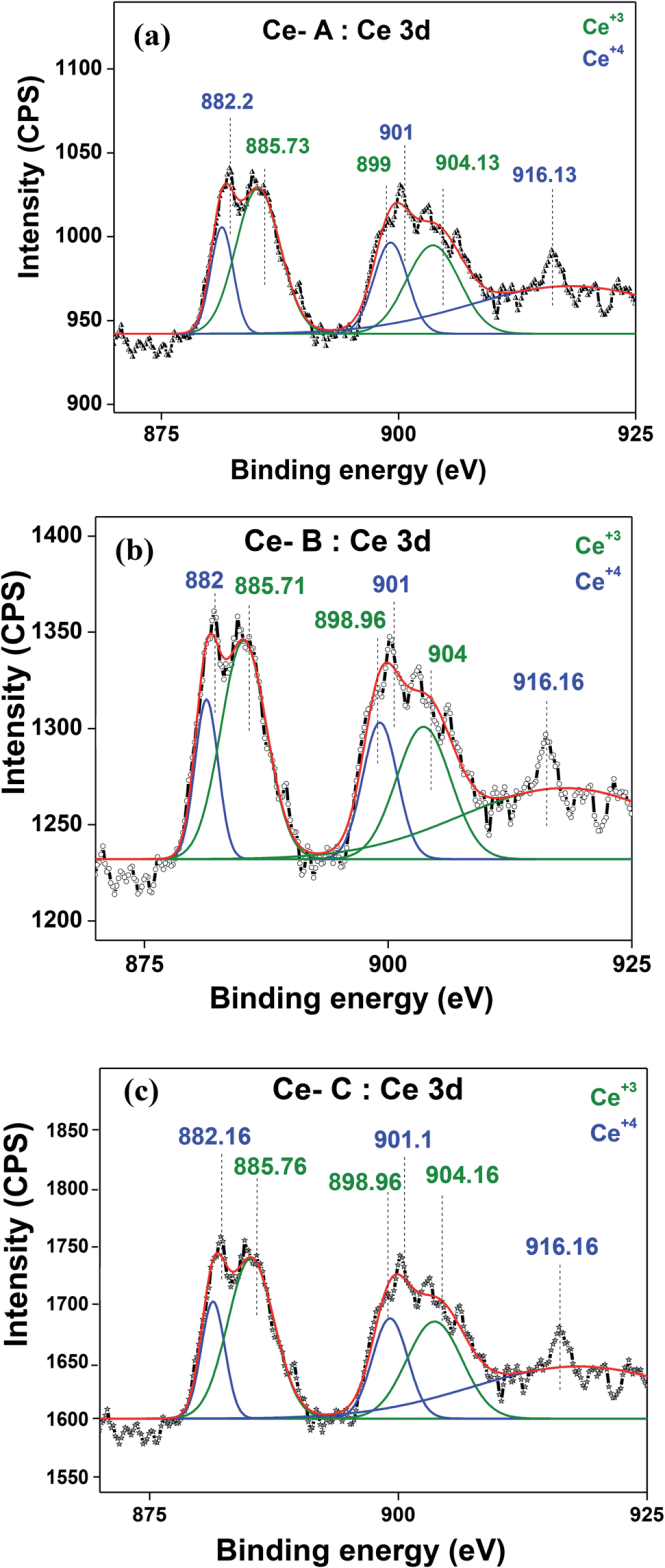
(a–c) Ce 3d core-level XPS peaks of Ce-A, Ce-B, and Ce-C, respectively.

**Fig. 6:**
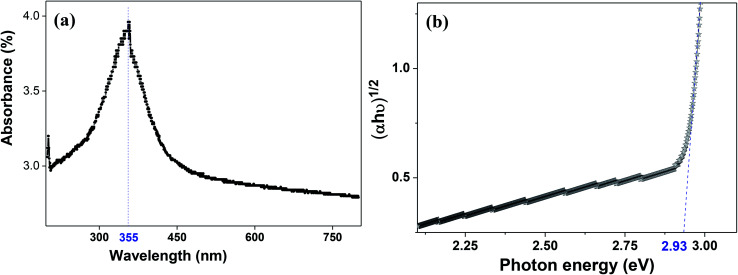
(a) UV-visible spectroscopy and (b) plot of (*αhν*)^1/2^*vs.* photon energy (eV) for Ce-C ceria flowerlike structures.

**Fig. 7:**
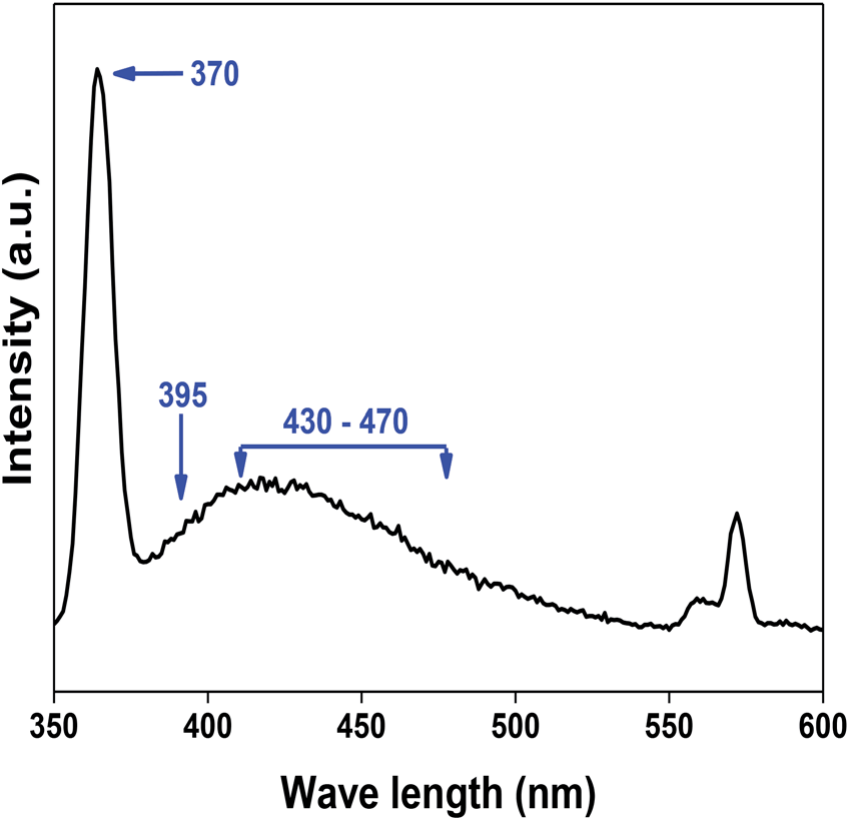
PL spectroscopic analysis of Ce-C ceria nanoflowers showing the characteristic wavelengths responsible for surface reactivity.

**Fig. 8:**
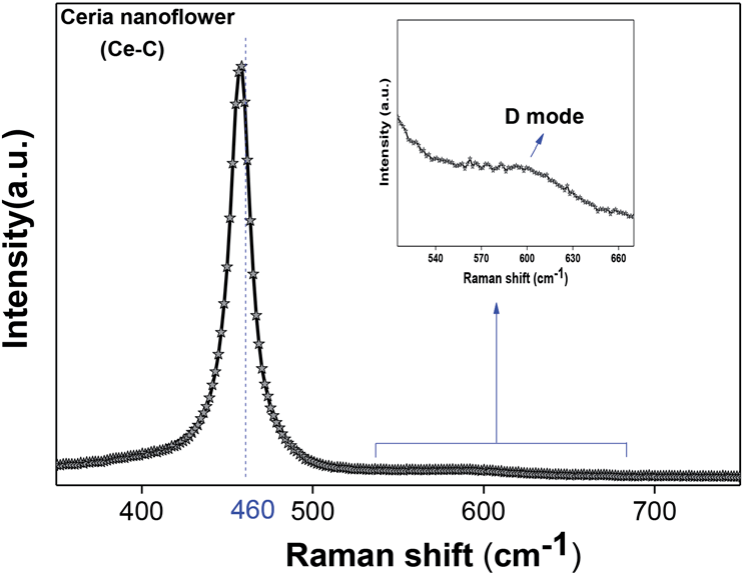
Raman spectroscopic analysis of Ce-C nanoflowers.

The molecular fingerprint of the final material was confirmed upon doing FTIR spectroscopic analysis. In Fig. S3,† the peak at 3470 cm^−1^ corresponds to the O–H stretching vibration of residual water and hydroxyl groups. The peak at 1624 cm^−1^ is due to the scissor bending mode of the associated water and the peak at 852 cm^−1^ corresponds to the Ce–O metal–oxygen bond. Other peaks at 1535, 1298, 1064 cm^−1^ belong to CeO_2_ nanoparticles. Besides this, elemental evidence of the occurrence of CeO_2_ is provided in the respective core level XPS analysis in Fig. 5. It shows the characteristic peaks of Ce^3+^ at 885.76, 898.96 and 904.16 eV and the other peaks at 882.16, 901 and 916.16 eV for the +4 oxidation state of CeO_2_.^38^ The flower-like structures provided an appreciable specific surface area to the synthesized CeO_2_ nanomaterials. Fig. 4(a)–(c) represents the nitrogen adsorption–desorption isotherms of Ce-A, Ce-B and Ce-C, respectively, including their pore size distributions. As expected, the BET specific surface area of Ce-C CeO_2_ nanoflowers was found to be appreciably high (∼132.69 cm^2^ g^−1^) as compared to that of Ce-A (∼54.03 cm^2^ g^−1^) and Ce-B (∼59.11 cm^2^ g^−1^). In the case of Ce-C, the higher surface area increased the areas of contact between the target analytes and the probability of surface reaction was increased as well. Thus, the rate of catalytic reaction was escalated. Prompted by the mesoporosity and high surface area, Ce-C CeO_2_ nanoflowers are expected to be a promising candidate for practical catalytic applications. The UV-vis absorption spectroscopic analysis of CeO_2_ nanoflowers is presented in Fig. 6. A strong absorption band in the UV region (∼355 nm) is clearly visible in Fig. 6(a) due to the charge transfer transitions from O 2p to Ce 4f bonds invading the f–f spin–orbit splitting of the Ce 4f state.^39^ The corresponding band gap of the material was calculated by plotting the Schuster–Kubelka–Munk absorption function, (*αhν*)^1/*n*^, against the photon energy (*hν*) according to the equation, (*αhν*)^1/*n*^ = *A*(*hν* − *E*). Where, *A*, *h*, *n*, and *α* represent the proportionality constant, Planck's constant, frequency of vibration, and absorption coefficient, respectively. Here, *n* = 2 for direct transitions. The band gap is 2.93 eV, calculated from the straight line *x*-intercept, presented in Fig. 6(b). Generally, the band gap of CeO_2_ is less than 3 eV. The narrowing of the band gap in CeO_2_ nanoflowers with appreciable surface area makes them a potential material for solid-state catalytic applications. The presence of oxygen vacancies, confirmed using PL spectroscopy (Fig. 7) shows the formation of oxygen vacancies in CeO_2_. Oxygen vacancies play a pivotal role in the narrowing of the band gap. It can better be represented by the Kröger–Vink equation, 
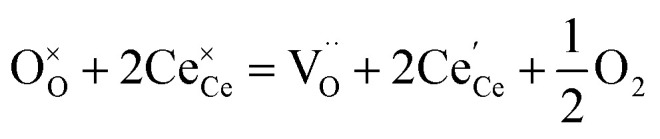
, where 
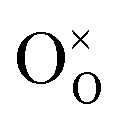
 is the O^2−^ ion on the oxygen lattice site; 
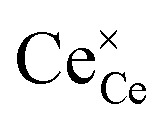
 and 
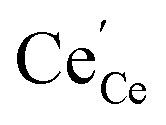
 denote Ce^4+^ and Ce^3+^, respectively; 
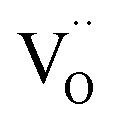
 is a doubly charged oxygen vacancy, and its formation emits two free electrons. The Ce^4+^ cations capture these electrons and are converted into Ce^3+^ ions. Due to this conversion, oxygen vacancies are developed, which lead to lattice distortion, causing the narrowing of the band gap. This shortening of the band gap is indeed a good feature for semiconducting materials for use in surface catalytic applications. PL emission occurs due to the transition from the 4f band of Ce to the 2p band of O, and the emission peaks are found in the range of 350 to 575 nm, as presented in Fig. 7. The CeO_2_ nanoparticles exhibit a strong emission peak at around 370 nm, which can be assigned to the hopping of electrons from the localized Ce 4f state to the O 2p valence band.^40^ Additionally, the appearance of a peak at around 395 nm is believed to originate from defect states that exist between the abovementioned states. The broad shoulder peak observed with much lower intensity than the first one in the range of 430–470 nm can be attributed to the presence of abundant defect states, helping in fast oxygen transport in the material. Besides, the quenched intensity of the shoulder peak somehow agrees with the longer charge separation, supporting the better surface reactivity of the CeO_2_ nanoparticles, the presence of oxygen defects and surface defects.^41^ The elemental evidence, the core-level Ce 3d spectra of Ce-A, Ce-B, and Ce-C in Fig. 5(a)–(c) provide strong insight on their oxygen vacancies as well.^42^ Here, in FCC-type nanostructures, a Ce^4+^ cation is always surrounded by eight O^2−^ ions. Reportedly, the coordination number of cerium is reduced from eight to seven in the presence of the oxygen vacancies and the accompanying Ce^3+^ and it affects the Ce–O bond length and overall lattice constant. This is in agreement with our structural parameter analysis (Table 1). As reported by Voskanyan *et al.*, a quantifiable approach was revealed regarding the XPS results, stating that CeO_2_ with a higher percentage of Ce^3+^ ions shows better catalytic activity.^43^ During the formation of oxygen vacancies, the oxygen leaves two excess electrons on Ce^4+^ cations, which fill the empty 4f orbitals of the Ce^4+^ and form Ce^3+^. Therefore, the quantification of Ce^3+^ could be treated as an effective parameter for the oxygen vacancy formation or the activity towards CO oxidation. In our case, the Ce-C ceria nanoflower [Fig. 5(c)] is found to contain a greater percentage of Ce^3+^ (∼28%) than rod-like Ce-B (∼13.06%) and lump-like Ce-A (∼12.76%) as shown in Fig. 5(b) and (a), respectively. Likewise, the signature of oxygen vacancies in different CeO_2_ structures can also be traced from their respective plots, as shown in Fig. S4.† XPS profiles of O 1s show a low binding energy peak at ∼529 eV (lattice oxygen) and a high binding energy peak at ∼532 eV (chemisorbed oxygen). Reportedly, chemisorbed oxygen is directly proportional to the associated oxygen vacancies and also increases their mobility over the catalysts.^44–47^ Therefore, there is an enhancement in the corresponding catalytic efficacies. Fig. S4(a)–(c)† furnish the chemisorbed oxygen of Ce-A, Ce-B and Ce-C, respectively. The percentage of chemisorbed oxygen was found to be ∼31.03% for Ce-C (Fig. S4c†), while the same, quantified in the case of Ce-A and Ce-B was ∼14.43% and ∼17.29, respectively. Therefore, in addition to the greater percentage of Ce^3+^, a greater amount of chemisorbed oxygen species shows the evolution of higher oxygen vacancies in Ce-C and substantiates the superior catalytic properties of Ce-C nanoflowers as compared to Ce-A and Ce-B. Raman spectroscopic analysis is another important tool regarding the study of oxygen vacancies. Fig. 8 represents the characteristic peak around 460 cm^−1^, which could be assigned to the Raman-active vibrational mode (F_2g_) of the CeO_2_ FCC structure. It occurs due to the symmetrical stretching vibration of the oxygen atoms around cerium ions.^48^ A shift of this peak toward lower frequency than 460 cm^−1^ may be correlated to asymmetry induced by randomly oriented oxygen vacancies, which expedites the associated catalytic activity. A weak band at around 600 cm^−1^ (inset) could be indexed to the defect-induced (D) mode of CeO_2_ nanostructures.^49^ Additionally, according to Askrabi *et al.*, the appearance of Raman modes at around ∼460 cm^−1^ is ascribed to the crystalline nature of CeO_2_ and corresponds to the symmetric “breathing” vibrations of the oxygen anions around the cerium cation.^50^ During the sonochemical reaction, considering the formation of CeO_2_, a liquid–solid heterogeneous system is formed. Ultrasound treatment facilitates mass transportation, causing particle fragmentation, and it is commonly associated with a large number of dangling bonds, defects or traps on their surface, increasing the surface reactivity of the material.

### 3.3. Plausible formation mechanism

**Scheme 1:**
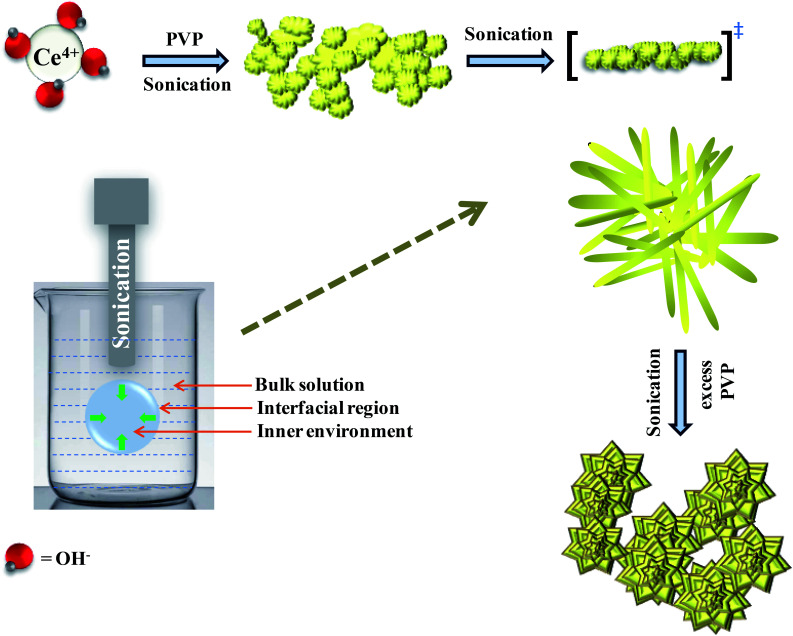
Plausible mechanistic pathway of CeO_2_ nanoflower formation.

Hydrolysis of cerium ammonium nitrate under sonication is the primary reaction, responsible for the occurrence of CeO_2_ nanoparticles. Now, following the morphological evidence and considering the influencing factors, the formation of CeO_2_ nanoflowers can be discussed. Initially, Ce^4+^ ions bearing a high charge concentration due to the inherent lanthanide contraction, readily undergo strong hydration to form [Ce(OH)_*x*_(H_2_O)_*y*_]^(4−*x*)+^. Under ultrasound, bubbles in the solution implosively collapsed *via* acoustic fields, and high-temperature and high-pressure fields were generated at the centres of those bubbles. Reportedly, the sonochemical process consists of three different regions: (i) the inner environment (gas phase) of the collapsing bubbles, where the elevated temperatures and pressures are generated and they lead to the formation of H˙ and OH˙; (ii) the interfacial region between the cavitation bubbles and the bulk solution, here, the temperature is lower than the aforesaid region; (iii) the bulk solution of ambient temperature. Considering the high quenching rate experienced by the products, it seems that the sonochemical reaction takes place at the interfacial region and CeO_2_ nanoparticles are obtained. If reactions take place in the gas phase, the local temperature depends on the vapour pressure of the solvents. Using water as a solvent, the maximum attainable temperature would be ∼4000 K and as a result of cooling during collapse, the obtained product would be amorphous in nature. Incidentally, reactions taking place within the interfacial region, at a temperature of ∼1900 K, would result in nanocrystalline products. In our case, the XRD (Fig. 1), Rietveld analysis for structural parameters, HRTEM and SAED (Fig. S1†) analysis showed the formation of crystalline product. These facts again second the formation of CeO_2_ nanoparticles within the interfacial region. The reactions occurring at the interfacial regions are reported to be influenced by the solvent and additive or surfactants present in the reaction mixture. Here, the surfactant PVP plays a pivotal role in the formation of rod-like CeO_2_ nanoparticles. In the reaction mixture, OH^−^ ions are adsorbed on the surface of tetravalent cerium under basic conditions. Subsequently, aggregation occurs due to the attraction, caused by the elimination of adjacent surface hydroxyl groups (*via* dehydration under sonication). Here, a precipitate could not be obtained even after the formation of CeO_2_ nanoparticles (as seen from the respective XRD data, Fig. 1) due to the presence of surfactant PVP. Thus, a colloidal solution (containing lump-like CeO_2_, Fig. 2(a)) was found and it continued to follow a growth mechanism under the influence of PVP in EG towards the formation of a flower-like 3D morphology. The PVP is adsorbed on the CeO_2_ nanoparticles through van der Waals attraction and direct binding. Under basic conditions, it becomes more favourable. PVP binds strongly to the {100} facets (lowest energy facet) to facilitate growth along the 〈111〉 direction (in agreement with XRD analysis (Fig. 1)). The associated peak intensity corresponding to this growth is greater for Ce-C as compared to Ce-A and Ce-B. A similar trend was found in the case of their structural lattice parameters, determined using Rietveld analysis. This growth of the plane under the influence of surfactants contributes to associated defect states as well.^51^ The increase in the cerium–oxygen bond length in Ce-C nanoflowers (as determined from Rietveld analysis in Table 1) confirms this phenomenon, leading to higher oxygen vacancies. Due to simultaneous reversible electron transfer between the nitrogen and oxygen atom, the PVP surfactant molecule suffers from a separation of charge, which consequently promotes its binding efficiency to respective oppositely charged surfaces in the reaction mixture. Additionally, PVP cannot form a branched structure as it consists of a five-membered ring with a nitrogen atom and an oxygen atom connected with a double bond to the ring. Thus, PVP molecules cover the metal oxide surface along one direction and facilitate the formation of rod-like structures (Fig. 2(b)). However, the occurrence of condensation/dehydration between surface hydroxyls under ultrasonic radiation outweighs the effect of steric hindrance, caused by the linear coverage of PVP molecules on the metal oxide surface. According to Banfield and his team, the adjacent nanoparticles spontaneously self-organize themselves to share a common crystallographic orientation, which is then followed by the joining of these particles in the same direction forming rod-like structures as shown in Fig. 2(c). During the attachment orientation, CeO_2_ nanoparticles were plausibly fused to each other by facets to reduce the total energy by removing the surface energy associated with unsatisfied bonds (Fig. 2(d)). After the addition of excess PVP, the growth along the previous direction became restricted; this further promoted the sidewise growth of the rods and resulted in the petal-like formation as shown in Fig. 2(e). An intermediate state of oriented petals towards fusion is incorporated in Fig. S5(a)† for a better understanding of the said morphology. The final morphology of cerium oxide (after 2 h of sonication) was found to be flower-like structures with tapered edges, as shown in Fig. 2(f). It is worth mentioning that with prolonged sonication up to 1 h, the structure of sidewise nanoflowers was fused as shown in Fig. S5(b).† This supports the tendency toward the fusion of single nanostructures in the reaction medium to form hierarchical 3D structures under the influence of sonication. Besides being a structure-directing agent, the PVP surfactant molecules around nanomorphologies play the role of interstitial filler and cause the assembly of ceria nanorods by the formation of intermolecular hydrogen bonding. PVP molecules not only directed the assembly of rod-like structures but also governed the formation of assemblies of rod-like/petal-like structures, resulting in 3D hierarchical flower-like structures with appreciable high surface areas and oxygen vacancies (supported by structural parameter analysis). van der Waals forces, electrostatic association with the aggregates, crystallization, hydrophobic interaction and hydrogen bond formation are also found to contribute to the flower-like structure formation. A pictorial representation of the process is included in the schematic diagram (Scheme 1).

## 4. Catalytic activity

**Fig. 9:**
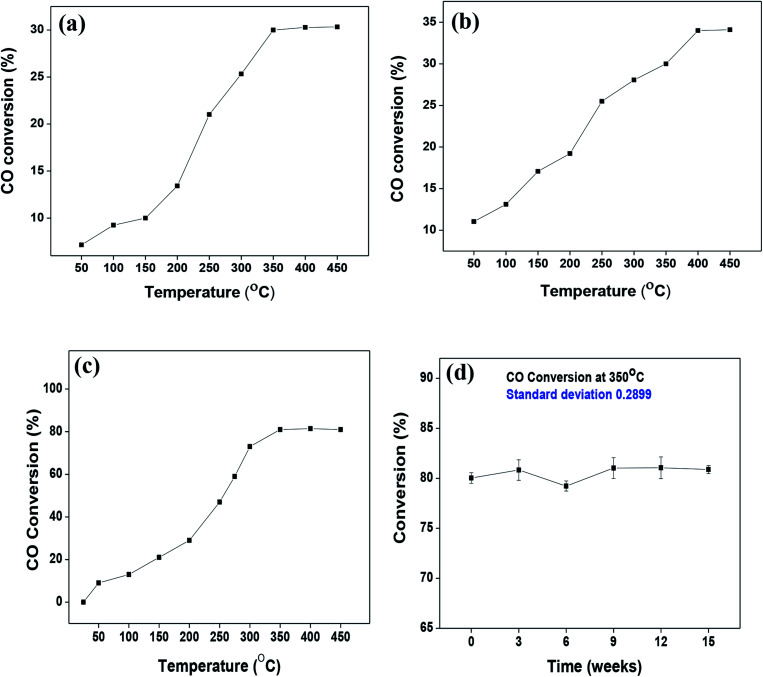
(a–c) Conversion of CO over Ce-A, Ce-B and Ce-C ceria structures, respectively, as a function of reaction temperature; (d) reproducibility/longevity study of the Ce-C catalyst nanoflowers as a function of time.

As mentioned previously, the surface catalytic reactivity of the CeO_2_ nanostructures was deployed for CO conversion. Fig. 9(a)–(c) represent the catalytic efficacies of Ce-A, Ce-B and Ce-C, respectively, as a function of temperature. The percentage of CO conversion seemed to increase with the increase in temperature from 25 °C to 400 °C under constant pressure for all the samples; however, their extents of conversion differed greatly. The CO conversion efficiencies of Ce-A (∼30%) and Ce-B (∼34%) were found to be notably lower than that of Ce-C (∼80%) CeO_2_ nanoflowers. As discussed previously, the low surface area (Fig. 4(a) and (b)) and associated lower oxygen vacancies (Fig. 5(a) and (b)) of Ce-A and Ce-B are majorly responsible for poor surface catalytic performances. Since, the surface area bears a strong correlation with surface defects, different surface areas of different structures influence the corresponding interaction strength with surface adsorbates and it affects the associated catalytic performance as well.^51^ In Fig. 9(c), the appreciably high conversion of CO at 350 °C in the presence CeO_2_ nanoflowers can be attributed to its appreciably high surface area and corresponding surface reactivity. The rate of conversion was found to increase with the increase in temperature up to 350 °C and beyond that, it attained saturation. To check the reproducibility of the Ce-C catalysts, the conversion efficiency was observed over a period of 15 weeks. Fig. 7(b) shows a steady performance of ∼80% conversion efficiency of the nanoflower catalyst with standard deviation even less than 1. Post-conversion morphology retention of the ceria nanoflower catalyst is included in Fig. S6.† The abundant oxygen vacancies of CeO_2_ (as confirmed from PL, XPS and Raman spectroscopies), huge oxygen storage capacity, facile transition between its Ce^3+^ and Ce^4+^ oxidation states and the appreciably high effective surface area of Ce-C 3D nanoflowers providing enhanced surface coverage of oxygen adsorbates promote the surface catalytic oxidation of CO. Generally, oxygen molecules are adsorbed on the metal oxide surface either dissociatively or nondissociatively in a random manner and result in the trapping of electrons from the conduction band and the formation of charged oxygen molecular species like O_ads_^2−^, O_ads_^−^, and O_2,ads_^−^. These adatoms remain electrostatically stabilized at the surface of the metal oxide and react with CO, which is reducing in nature. This phenomenon takes place in the temperature range of 100–500 °C. In our case, it was likely to occur during the conversion around 350°, leading to an increase in conversion efficiency up to 80%. Initially, the physisorption of CO on 3D nanoflowers occurred *via* dipole bonding to the metal-oxide–semiconductor surface, unfastening the electrons from high oxygen vacancy, and subsequently, charge transfer resulted in the formation of a chemical bond with the surface atoms, commonly termed as chemisorption. The beneficial monolayer adsorption of CO (concluded from the adsorption isotherm, Fig. 4(c)) on the surface of the ceria nanoflowers facilitates the reaction even at low temperature (50–100 °C). It may be noted that during the physical adsorption, depending on gas concentration, there may be the formation of both multilayers and monolayers. A comparative performance analysis is included in Table 2, representing the catalytic activities of different morphologies of CeO_2_ nanostructures towards CO oxidation. It also shows that the Ce-C, CeO_2_ nanoflower has the lowest synthesis temperature and simplest technique, providing comparably appreciable catalytic efficiency towards CO oxidation as per our knowledge.^52–54^ The above observations suggest that the Ce-C ceria 3D nanoflowers with high effective surface areas are a potential candidate for use as material in CO conversion with appreciable efficiency over a long period.

**Table 2:** Comparative performance analysis of pure CeO_2_-based catalysts towards CO oxidation

CeO_2_ morphology	Synthesis temperature (°C)	Conversion efficiency	Conversion temperature (°C)	Reference
Flower-like	Room temperature, 2 h	80%	350	This article
Nanorods	100 °C, 72 h	50%	224	52
Nanoparticle	110 °C, 24 h	50%	305	52
Nanowires	120 °C, 24 h	50%	245	52
Nanotube	120 °C, 72 h	50%	223	52
Nanocube	180 °C, 24 h	50%	315	52
Nanorods	180 °C, 24 h	20%	220	53
Nanoflower	180 °C, 24 h	50%	250	54
Commercial CeO_2_ catalyst	—	90%	320	54

## 5. Conclusion

The present article delineates a simple room-temperature synthesis of mesoporous 3D CeO_2_ nanoflowers with appreciable surface area *via* sonication. The structure-directing efficacy of PVP is widely explored for tweaking the morphological parameters. The properties of the 3D nanoflower catalyst are discussed with plausible mechanisms and structural parameter analysis. The surface catalytic reactivity of ceria nanoflowers is attributed to their corresponding physicochemical properties and high surface area. The CO conversion capacity, as well as their long term stability and sustainability, has also been tested. Preliminary catalysis evaluation shows the 3D CeO_2_ nanoflowers to be an effective catalyst for CO oxidation. Moreover, this synthetic strategy may become a useful environmentally benign method for the scaling-up of ceria-based catalysts.

## Conflicts of interest

There are no conflicts to declare.

## Supplementary Material

RA-010-D0RA02554B-s001
