# Supplementary material for: Room temperature blooming of CeO2 3D nanoflowers under sonication and catalytic efficacy towards CO conversion
Source: RSC Adv. 2020 Jun 9;10(37):22204–15. doi: 10.1039/d0ra02554b (PMC9054551; doi:10.1039/d0ra02554b)
Supplement: RA-010-D0RA02554B-s001 [file RA-010-D0RA02554B-s001.pdf]

Electronic Supplementary Material (ESI) for RSC Advances.
This journal is © The Royal Society of Chemistry 2020

Room Temperature Blooming of CeO_2 3D Nanoflowers under Sonication and Catalytic Efficacy towards CO Conversion

Deblina Majumder,*[a] Indranil Chakraborty[a], Kalyan Mandal[a]

[a]S. N. Bose National Centre for Basic Sciences, Block JD, Sector III, Salt Lake, Kolkata 700106, India

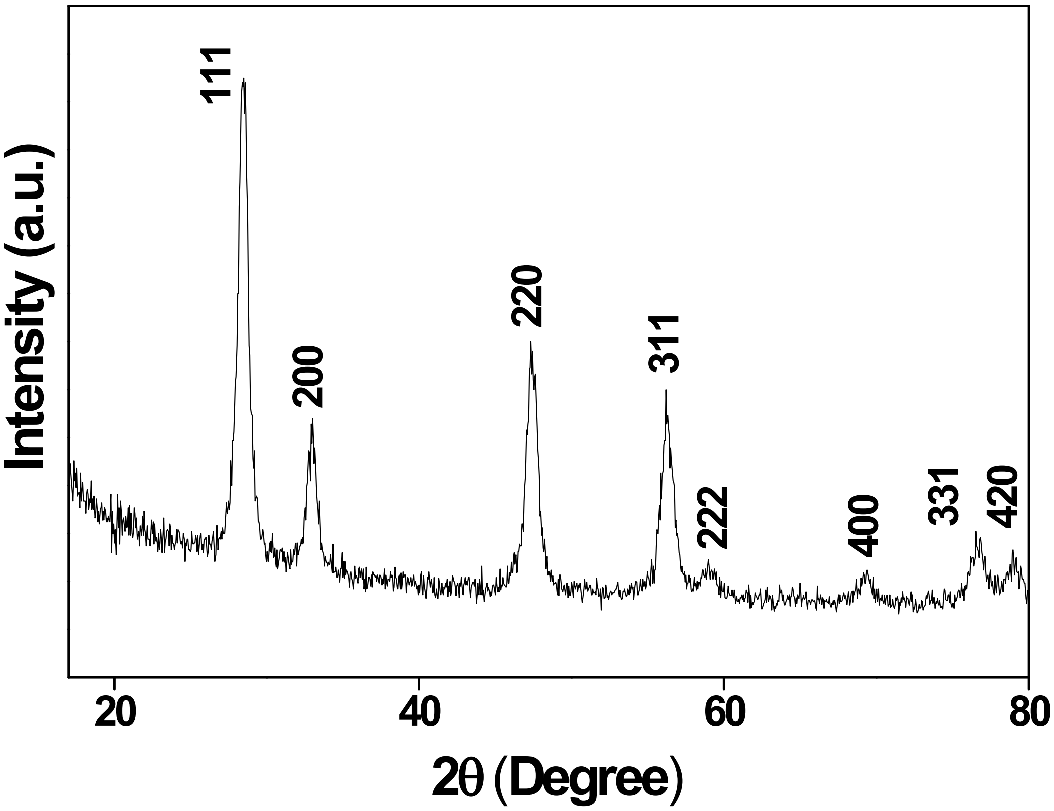

Figure S1: Post calcination XRD plot of Ce-C CeO_2 nanoflower

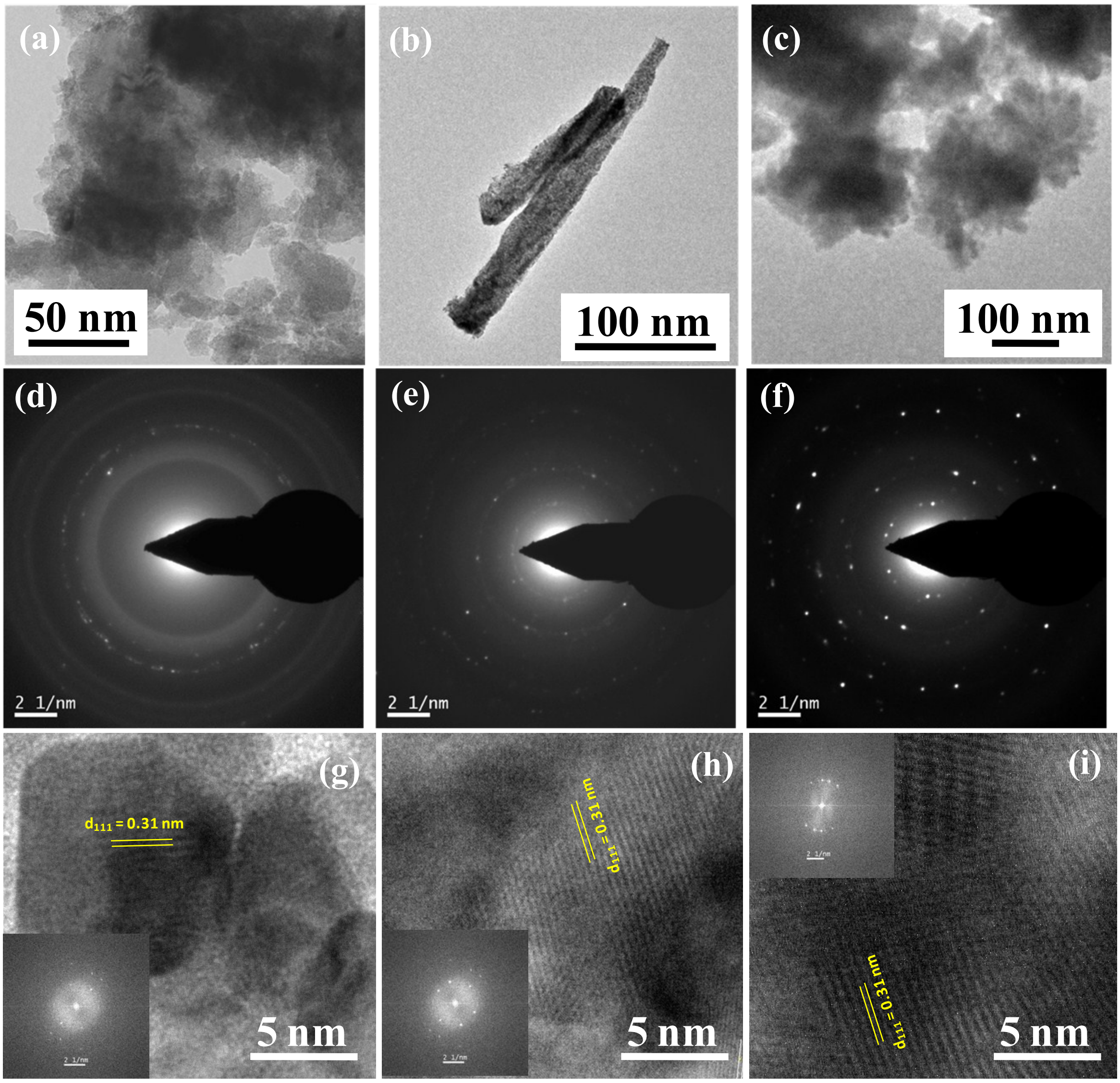

Figure S2: (a)-(c) TEM micrographs, (d)-(f) corresponding SAED pattern and (g)-(i) HRTEM (inset) of Ce-A, Ce-B and Ce-C respectively.

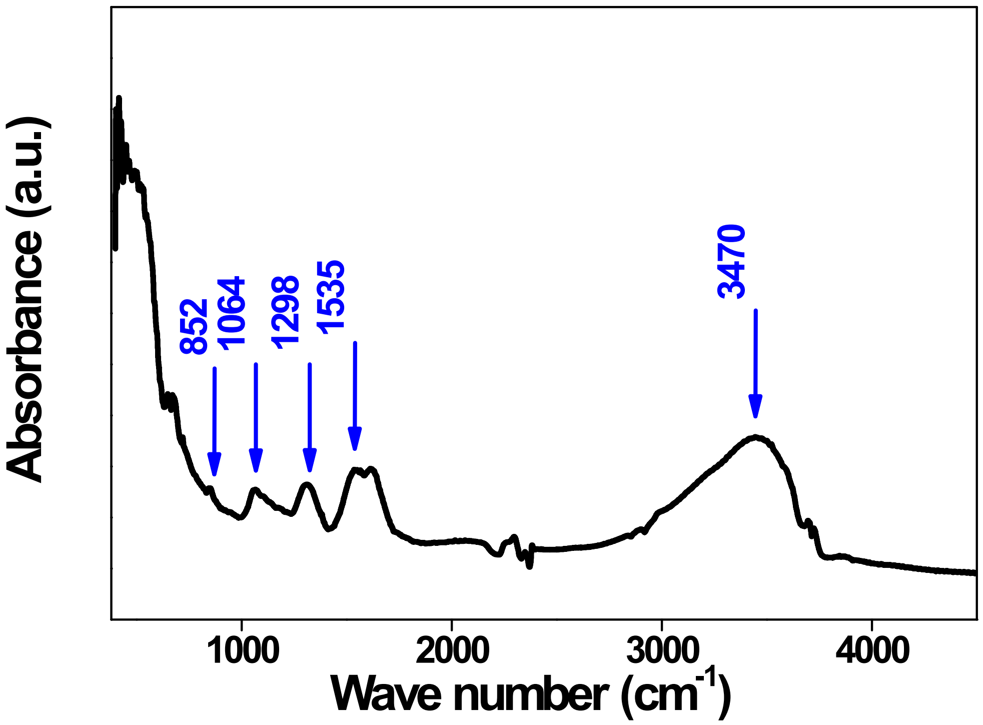

Figure S3: FTIR spectroscopy of ceria nanoflower, Ce-C.

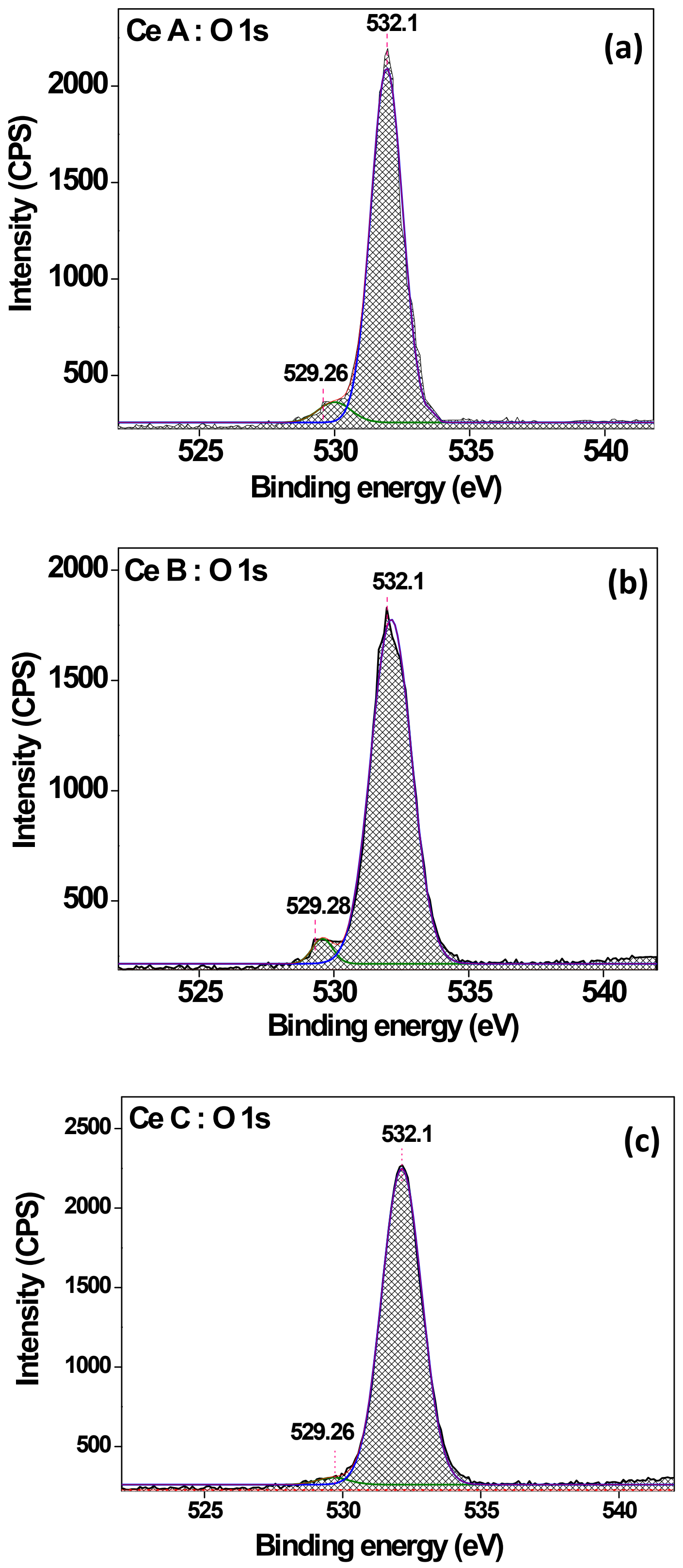

Figure S4: (a)-(c) O 1s core level XPS spectra of the Ce-A, Ce-B and Ce-C respectively.

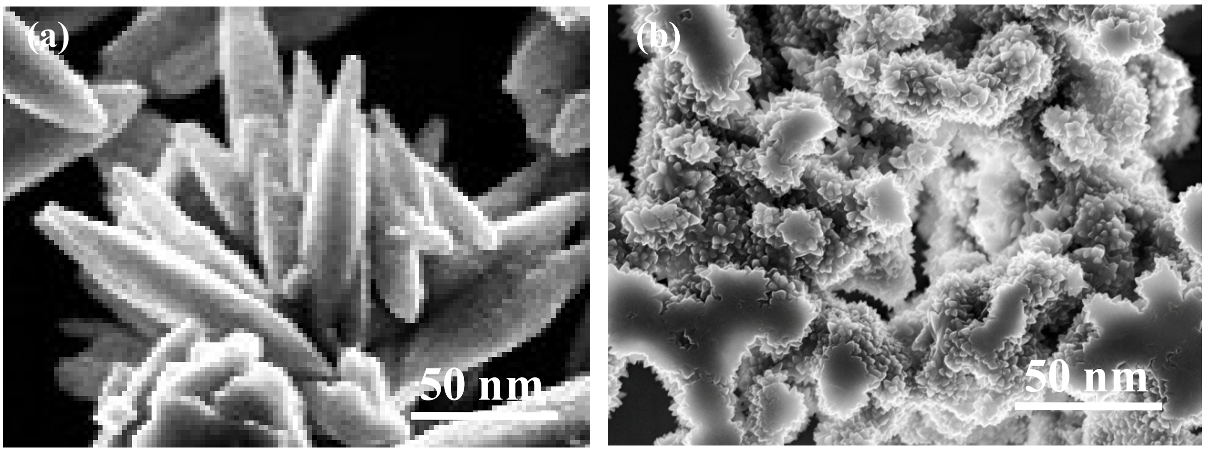

Figure S5: (a) intermediate state of formation of flowerlike structure, (b) prolonged sonication beyond 2 h, fused flowerlike structure.

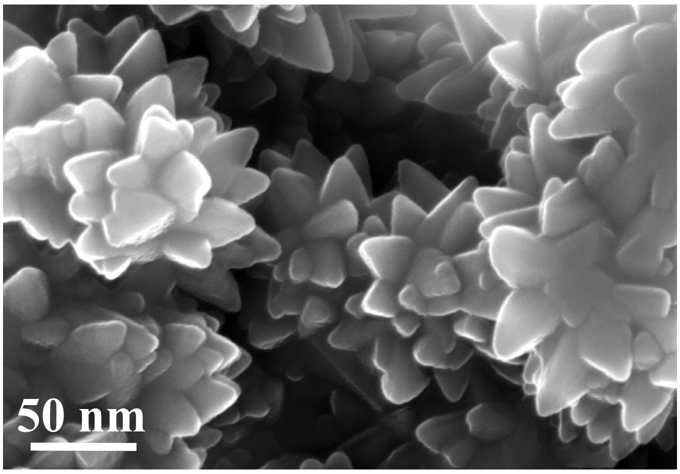

Figure S6: Post conversion FESEM image of Ce-C ceria nanoflower indicating no morphological or structural change
